# Incidental findings in cone beam computed tomography (CBCT) scans for implant treatment planning: a retrospective study of 404 CBCT scans

**DOI:** 10.1007/s11282-023-00723-5

**Published:** 2023-12-15

**Authors:** Philippe Biel, Alice Jurt, Vivianne Chappuis, Valerie G. A. Suter

**Affiliations:** Department of Oral Surgery and Stomatology, School of Dental Medicine, University of Bern, Freiburgstrasse 7, CH-3010 Bern, Switzerland

**Keywords:** Cone beam computed tomography, Dental implant planning, Incidental finding, Field of view, Maxillofacial radiology

## Abstract

**Objectives:**

To investigate the prevalence of incidental findings and need for further dental treatment and analyse the influence of size of field-of-view (FOV) and age in cone beam computed tomography (CBCT) for pre-implant planning.

**Methods:**

404 CBCT scans were examined retrospectively for incidental findings and need for further dental treatment. Incidental finding-frequencies and need for further treatment were assessed for different age (< 40 years, 40–60 years, > 60 years) and FOV groups (small, medium, large). Intraexaminer and interexaminer agreements were evaluated.

**Results:**

In 82% of the scans at least one incidental finding was found, with a total of 766 overall. More incidental findings were found in scans with large FOV (98% vs. 72%, OR = 22.39 large vs. small FOV, *p* < 0.0001) and in scans of patients > 60 years (OR = 5.37 patient’s age > 60 years vs. < 40 years, *p* = 0.0003). Further dental treatment due to incidental findings was needed in 31%. Scans with large FOV were more likely to entail further treatment (OR = 3.55 large vs. small FOV, *p* < 0.0001). Partial edentulism and large FOV were identified as risk factors for further treatment (*p* = 0.0003 and *p* < 0.0001). Further referral of the patient based on incidental findings was judged as indicated in 5%. Intra- and inter-examiner agreements were excellent (kappa = 0.944/0.805).

**Conclusions:**

A considerable number of incidental findings with need for further dental treatment was found in partially edentulous patients and in patients > 60 years. In pre-implant planning of elderly patients, the selection of large FOV CBCT scans, including dentoalveolar regions not X-rayed recently, help to detect therapeutically relevant incidental findings.

## Introduction

Cone beam computed tomography (CBCT) was introduced in the 1980s in angiography [1]. After being used for the first time in dentistry in 1998 and becoming commercially available, CBCT gained a wide acceptance with a rapid evolution of the technology [2]. Due to its precise multiplanar reconstruction with a high resolution, CBCT is today widely used in dentistry for diagnostics and the assessment of bone morphology and anatomic structures of the jaws, especially in implant treatment planning [3–7].

Compared to conventional computed tomography (CT), scan time and radiation dose for image acquisition are significantly lower with CBCT [8]. Most CBCT machines allow the examination of parts of the oral-maxillofacial region with small field-of-views (FOVs) and reduce unnecessary radiation to dentomaxillofacial regions and sensitive tissues, like bone marrow, salivary glands and oral mucosa [9]. According to the as low as reasonably achievable (ALARA) principle to reduce the effective radiation dose, small FOVs are chosen in CBCT image acquisition whenever possible [10].

On the other hand, it has been shown that incidental findings are found in more than 90% of CBCT scans with large FOVs [6, 11–14]. Such an incidental finding can be defined as “an occult entity discovered unexpectedly on an imaging examination performed for an unrelated reason” [15]. The dentist acquiring the CBCT has to define or verify the indication, select the dimension of the FOV and analyse and interpret the whole volume. If the responsible dentist is not trained in CBCT diagnostics, he should refer the patient to an oral and maxillofacial radiologist to interpret the CBCT in order to detect these incidental findings and provide the patient with optimal treatment in a timely manner [16].

In the current literature, four studies describe the analysis of incidental findings in CBCT scans with large FOV made only for dental implant treatment planning [6, 12, 17, 18]. And, to the best of our knowledge, there are only a few studies that assessed the prevalence of incidental findings in small FOV CBCTs, and these studies were performed mainly for other dental purposes than implant planning, for example, impacted canines [19] and endodontics [20]. The influence of the dimension of the FOV and age on the prevalence of incidental findings has hardly been studied yet. Moreover, there is no generally established recommendation on the ideal size of FOV for CBCT scans for implant pre-treatment examination despite a suggestion of a recently published study to acquire large FOV (10 × 10 cm) CBCT for implant treatment planning [21].

The aim of this retrospective study was to assess the prevalence and type of incidental findings depending on the size of FOV and patient’s age in CBCT scans acquired for dental implant pre-treatment examination and planning. Secondarily, the frequency of radiologically judged need for further dental treatment due to incidental findings found in implant pre-treatment examination was investigated.

## Materials and methods

### Study design

The study protocol was approved by the standing ethics committee of the State of Bern and by Swiss Ethics (Project-ID 2021-00920). All patients whose CBCT scans have been included in the study have signed the general consent of the School of Dental Medicine, University of Bern, and approved that their data can be used. A radiology technician not involved in the study checked all radiology reports of CBCT scans of patients referred for a CBCT examination to the Section of Dental Radiology and Stomatology in the Department of Oral Surgery and Stomatology at University of Bern from 31st December 2020 backwards to specify the reason of referral. The first 420 CBCT scans of patients referred for dental implant treatment planning were included in the study and retrospectively re-examined for incidental findings. These scans were acquired between 02.07.2018 and 14.12.2020. CBCT scans from patients referred for another reason than dental implant treatment planning, for dental implant treatment planning combined with another diagnostic question and scans that presented a remaining tooth or an already placed implant in the planned implant site were excluded. In addition, all patients referred from a maxillofacial surgery clinic were excluded, as they were highly suspected to have suffered a trauma or previous surgery that may be a bias in evaluating incidental findings. The 420 included CBCT scans were subsequently examined systematically for incidental findings by one observer (PB) with 2 years of experience in CBCT diagnostics and reporting at the department. No previous radiograph or documents from clinical examination were accessible to the observer. PB was not involved in the initial CBCT examination or patient’s treatment planning or treatment. Of the initially included 420 CBCT scans, 16 cases were excluded after re-examination of the CBCT scans, because implants were already placed in the planned implant site (*n* = 3), teeth were still present in the planned implant site region (*n* = 11) or another diagnostic question was in the referral (*n* = 2). Finally, 404 CBCT scans were included for the analysis. To check intraobserver reliability, 50 randomly chosen CBCT scans out of the included 404 were re-examined by the first observer (PB) after a 1-month period. To check interobserver reliability, a second observer (VS) with over 15 years of experience of CBCT diagnostics and reporting examined another 50 randomly selected CBCT scans.

### Image acquisition

Patients were sent to the Section of Dental Radiology and Stomatology in the Department of Oral Surgery and Stomatology with a referral containing a diagnostic question and the region of interest to be imaged for CBCT imaging and were not clinically examined. The dimension of the FOV was set by the Head of the Section with the smallest possible FOV to answer the diagnostic question in the referral. All CBCT images were acquired with the same machine, a 3D Accuitomo 170 unit (Morita Corp., Kyoto, Japan). Patients were sitting, the head fixed and the horizontal position aligned to the lower border of orbita to tragion and centered in the middle of the face for the sagittal. Most (*n* = 400) CBCT images were obtained with full (360°) scan rotation for 17.5 s (standard mode), one with 10.5 s (high-speed mode), one with 30.8 s (high resolution mode) and two with half (180°) scan rotation for 9 s. Exposure settings of all scans were 5.0 mA and 90 kV. The FOV and respective voxel size was 4 × 4 cm/80 μm (*n* = 106), 6 × 4 cm/125 μm (*n* = 46), 6 × 5 cm/125 μm (*n* = 74), 6 × 6 cm/125 μm (*n* = 4), 8 × 4 cm/160 μm (*n* = 5), 8 × 5 cm/160 μm (*n* = 58), 8 × 8 cm/160 μm (*n* = 22), 10 × 5 cm/160 μm (*n* = 31), 10 × 10 cm/160 μm (*n* = 56), 14 × 5 cm/160 μm (*n* = 1) or 14 × 10 cm/160 μm (*n* = 1). The CBCT scans were evaluated within the software program i-Dixel (Morita Corp., Kyoto, Japan) on a Dell Precision 7820 workstation (Dell, Round Rock, Texas, USA) with a 19-inch Eizo Flexscan monitor (resolution of 1280 × 1024 pixels; Eizo Nanao AG, Wädenswil, Switzerland).

### Outcome measures and data collection

First, an enumeration and group classification of incidental findings has been made based on former studies on incidental findings in CBCT and according to the authors’ experience with slight modifications and add-ons [6, 7, 17, 22, 23]. The two observers then evaluated the entire FOV of each or randomly selected (2nd observer, VS) CBCT scan for any incidental finding. They had also to decide if they assumed further imaging with a CT scan, magnetic resonance imaging (MRI) or ultrasonography (US) were necessary for diagnostics. Additionally, the two observers assessed whether further dental treatment was indicated based on the incidental finding (yes or no). Additionally, it was assessed if a referral to an otorhinolaryngologist or maxillofacial surgeon was necessary. Patient-related data (age, gender), displayed region of the jaw in the FOV (maxilla, mandible, maxilla and mandible) and number and localization of missing teeth in the FOV were also collected. Image acquisition data, patient-related data and observer’s data were recorded in an electronic research data capture tool (REDCap) [24].

### Statistical analysis

To set the sample size of CBCT scans included in the study, a power analysis was carried out for the primary outcome, i.e. the frequency of incidental findings depending on FOV size. According to the power analysis, a sample size of *n* = 400 was required. 420 scans were selected for further evaluation and 16 cases were excluded, resulting in a total of 404 scans for statistical assessment.

Descriptive statistics, including the frequency of incidental findings and the need for further dental treatment were calculated. Three age groups were created (< 40 years, 40–60 years, > 60 years). For further analysis of the incidental findings, three groups were formed depending on the dimension of FOV: small FOV (4 × 4 cm, 6 × 4 cm, 6 × 5 cm), medium FOV (6 × 6 cm, 8 × 4 cm, 8 × 5 cm, 8 × 8 cm, 10 × 5 cm) and large FOV (10 × 10 cm, 14 × 5 cm, 14 × 10 cm).

The influence of the dimension of FOV, age, tooth status and gender on incidental finding frequencies (binary, “yes” or “no”) in general and regarding incidental finding types as well as on the need for further dental treatment was assessed using Fisher’s exact test and post hoc test in case of two categories or using an extended bootstrap version in case of three categories. Logistic regression models were used to confirm significant factors in a multivariate analysis. Intraexaminer and interexaminer agreements were assessed using Cohen’s unweighted kappa value for two raters, with 50 replicas in each case (kappa < 0.4 = poor; 0.4–0.6 = fair; 0.6–0.75 = good; > 0.75 = excellent) [25].

The level of significance was set at 0.05. Post hoc tests were corrected by the method of “Holm”. All results were calculated using the statistical software R, version 4.0.2 [26].

## Results

Patients (*n* = 404) were aged between 19 and 93 years with a median of 64 years and a mean of 62.6 years. The number of patients in the different age groups and the gender distribution are presented in Table 1. Regarding tooth status, 92% (*n* = 370) of the patients were partially edentulous, whereas 8% (*n* = 34) were fully edentulous in the imaged region. The partially edentulous patients had in 31% (*n* = 125) a single tooth gap, in 17% (*n* = 70), a multi-unit gap, in 25% (*n* = 99), a free-end situation and in 19% (*n* = 76), a combined situation. Most CBCT scans imaged the maxilla only (49%), followed by the mandible only (31%), and both the maxilla and mandible (20%) (Table 1). In total, 1972 teeth were missing in all 404 CBCT scans, corresponding to an average of 4.9 teeth per patient in the respective FOV.

**Table 1:** Demographics, imaged region, tooth status and dimension of FOV

Variable	Subgroup	Number of patients	% of patients
Age	min. 19 years, max. 93 years, median 64 years, mean 62.6 years		
	< 40 years	28	7
	40–60 years	115	29
	> 60 years	261	64
Gender	Female	218	54
	Male	186	46
Imaged region	Maxilla	199	49
	Mandible	126	31
	Maxilla and mandible	79	20
Tooth status	Partially edentulous		
	Single tooth gap	125	31
	Multi-unit gap	70	17
	Free-end situation	99	25
	Combined	76	19
	Fully edentulous	34	8
FOV	Small (4 × 4 cm, 6 × 4 cm, 6 × 5 cm)	226	56
	Medium (6 × 6 cm, 8 × 4 cm, 8 × 5 cm, 8 × 8 cm, 10 × 5 cm)	120	30
	Large (10 × 10 cm, 14 × 5 cm, 14 × 10 cm)	58	14

**Fig. 1:**
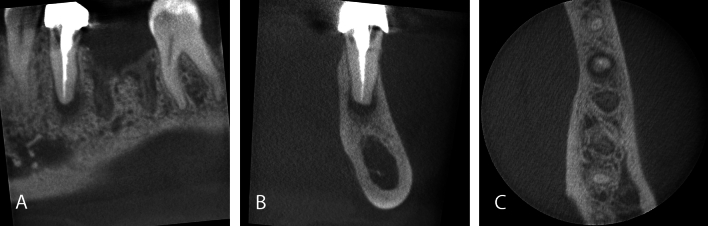
Small volume (4 × 4cm) CBCT scan of a 36-year-old male patient for implant pre-treatment examination in lower first molar position. A periapical radiolucency (apical lesion) of the root-treated neighbouring second premolar was identified as incidental finding. Sagittal (**A**), coronal (**B**) and axial (**C**) image

**Fig. 2:**
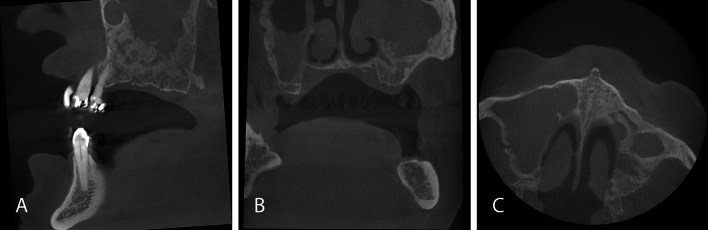
CBCT scan for digital implant planning with large FOV (10 × 10 cm) in a partially edentulous 79-year-old male patient. Opacification (over 50%) of the partially imaged left maxillary sinus antrum with inhomogeneous osseous appositions basally and absence of the left lateral nasal wall is conspicuous. In the right maxillary sinus, an opacification was present in the lower half of the antrum. Due to the incidental findings in the maxillary sinuses, further evaluation by an otorhinolaryngologist was indicated. Sagittal (**A**), coronal (**B**) and axial (**C**) images

**Fig. 3:**
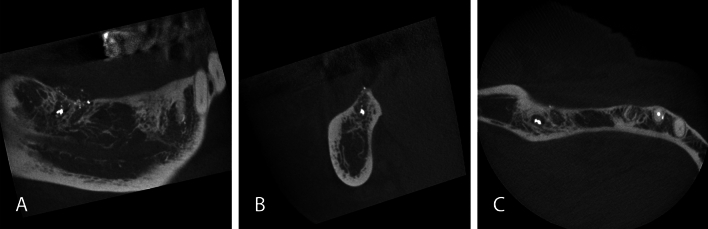
CBCT scan of a 71-year-old female patient with a small FOV (6 × 4 cm) for implant pre-treatment examination in free-end posterior right lower jaw. In the lower right wisdom tooth position a radiopaque concrement of dental filling material with circumferential radiolucency is visible. Furthermore, multiple small radiopaque concrements are recognizable in the residual socket and adjacent to the bone in the soft tissue (fragmented dental material in bone/in soft tissues). In the axial plane dish-like root remnants are identifiable in second premolar site, where implant placement was planned. Sagittal (**A**), coronal (**B**) and axial (**C**) images

**Fig. 4:**
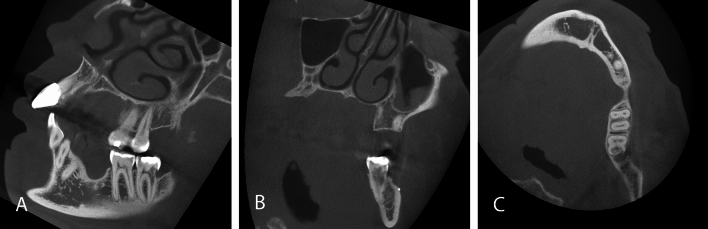
CBCT scan of a 41-year-old female patient with a large FOV (10 × 10 cm) with multi-unit gaps. A cervical root resorption was discovered as an incidental finding on the second lower left molar. Furthermore, in the coronal plane (**B**) a small radiopaque concrement is visible on the buccal bone as well as circumferential thickening of the maxillary sinus mucosa in the left maxillary sinus. Sagittal (**A**), coronal (**B**) and axial (**C**) images

A total of 766 incidental findings were found. In 82% (*n* = 330) of the scans, at least one incidental finding was found (mean incidental findings of 1.9 per scan, median of 2 per scan). The maximum incidental findings in one scan (FOV: 10 × 10 cm) was nine. The most common incidental finding was a thickening of maxillary sinus mucosa (basal flat or dome-shaped or circumferential flat or irregular thickening, *n* = 130), followed by fragmented dental material in bone or soft tissues (*n* = 118) and apical lesion (< 5 mm radiolucency, *n* = 72). Detailed enumeration and classification of incidental findings are presented in Table 2. Figures 1, 2, 3 and 4 show representative incidental findings found in four different CBCT scans.

**Table 2:** Enumeration and classification of incidental findings, need for further diagnostic assessment, referral or dental treatment

Type of incidental finding	Number of patients with incidental finding	% of patients with incidental finding
Maxillary sinus		
Basal flat thickening sinus mucosa > 2mm	91	23
Dome-shaped (semi-aspherical, cystic) thickening of sinus mucosa	9	2
Circumferential flat thickening or irregular thickening of sinus mucosa	30	7
Intramural calcifications	15	4
Iatrogenic bone defect, oroantral communication	6	2
Fluid accumulation, air bubbles in the maxillary sinus	5	1
Opacification over 50% (regardless of the shape of membrane thickening)	11	3
Destructive lesion/malignom	0	0
Blocked osteomeatal complex	2	1
Foreign body or tooth in the maxillary sinus	7	2
Alveolar or tuber extension of the maxillary sinus	1	0.2
Others	5	1
Implant apical in maxillary sinus	2	
Meatotomy	2	
Sinus floor elevation	1	
Other paranasal cavities (sinus frontalis, sinus sphenoidalis, cellulae ethmoidales)		
Thickening of sinus mucosa (> 2 mm)	4	1
Total opacification of sinus	0	0
Nasal		
Concha/turbinate hypertrophy	0	0
Concha bullosa	3	1
Nasal polyposis	1	0.2
Deviated septum	38	9
Others	1	0.2
Interrupted septum	1	
Bone		
Exostosis maxilla and/or torus palatinus	0	0
Exostosis/torus mandibularis	3	1
Bone sclerosis (idiopathic)	40	10
Malignant tumor	0	0
Others	3	1
Bone cavity/expanded bone marrow spaces	2	
Pins in bone	1	
Cystic		
Radicular cyst (> 5 mm radiolucency, distance measured from apex)	2	1
Dentigerous cyst/follicular cyst (> 5 mm follicular space)	0	0
Other odontogenic cyst	5	1
Nasopalatine cyst	2	1
Dentoalveolar/teeth		
Apical lesion (< 5 mm radiolucency, distance measured from apex)	72	18
Apicomarginal (endoperio) lesion	21	5
Condensing/sclerosing osteitis	14	4
Osteomyelitis/osteonecrosis	1	0.2
Sequestrum in extraction site (not related to osteonecrosis)	8	2
Overpressed root filling material (adjacent to root)	41	10
Fragmented dental material in bone/in soft tissues	118	29
External/cervical root resorption	18	5
Dens invaginatus	0	0
Residual root/root fragments	37	9
Impacted third molar	11	3
Impacted canine	2	1
Odontoma	0	0
Internal granuloma	1	0.2
Vertical bone loss (≥ 5mm)	64	16
Root fracture	9	2
Others	11	3
Very short roots	2	
Fractured instrument in root canal	1	
Angular root defect/root decay	6	
Periodontal space enlargement/occlusal trauma	1	
Peri-implantitis	1	
Soft-tissue calcifications		
Calcification of stylohyoid ligament or other ligaments	10	3
Tonsillolith	38	9
Sialolith	0	0
Calcified lymph node	1	0.2
Calcified pineal gland	0	0
Others	3	1
Foreign material in chin	2	
Seeds in soft-tissue	1	
Vertebral		
Degenerative change	0	0
Others	0	0
Vascular		
Calcification of atherosclerotic plaques in the carotid arteries	0	0
Others	0	0
TMJ		
Osteophytes	0	0
Flattening condyle	1	0.2
Condylar erosion	1	0.2
Condylar degenerative change	0	0
Subcondylar cyst	0	0
Bifid condyle	0	0
Others	0	0
Further diagnostic assessment (MRI, CT, US)	1	0.2
Further referral		
Referral to maxillofacial surgeon	0	0
Referral to otorhinolaryngologist	19	5
Further dental treatment needed	126	31

In female patients, an incidental finding was found in 83% of the scans and in male patients in 81% (Table 3). On the occurrence of any incidental finding in the CBCT scans, the patients’ gender had no significant influence. However, in male patients significantly more soft-tissue calcifications as incidental findings were detected than in females (odds ratio (OR) = 2.67, *p* = 0.004) (Table 4).

**Table 3:** Number of incidental findings and need for further dental treatment by groups

Category	Total number of scans	Number of examinations with incidental finding	Number of incidental finding per scan mean (sd)	Number of incidental finding per scan median (Q1, Q3)	Number of examinations with need for further dental treatment
Gender					
Female	218	180 (83%)	1.88 (1.61)	2 (1;2)	65 (30%)
Male	186	150 (81%)	1.92 (1.61)	2 (1;3)	61 (33%)
Age					
< 40 years	28	15 (54%)	1.43 (1.95)	1 (0; 2)	5 (18%)
40–60 years	115	90 (78%)	1.61 (1.32)	1 (1; 2)	33 (29%)
> 60 years	261	225 (86%)	2.07 (1.67)	2 (1; 3)	88 (34%)
Tooth status					
Partially edentulous	370	302 (82%)	1.93 (1.64)	2 (1; 3)	123 (33%)
Fully edentulous	34	28 (82%)	1.50 (1.14)	1 (1; 2)	3 (9%)
FOV					
Small	226	162 (72%)	1.19 (1.06)	1 (0; 2)	58 (26%)
Medium	120	111 (93%)	2.33 (1.49)	2 (1; 3)	36 (30%)
Large	58	57 (98%)	3.78 (1.84)	4 (2; 5)	32 (55%)

Number of scans and distribution of incidental findings and need for further dental treatment depending on patient’s gender (female, male) and age (< 40 years, 40–60 years, > 60 years), tooth status (partially edentulous, fully edentulous) and dimension of FOV (small FOV (< 6 × 6 cm), medium FOV (6 × 6 cm ≤ 8 × 8 cm, 10 × 5cm), large FOV (> 8 × 8 cm))

**Table 4:** Statistical analysis of incidental findings (IF) and need for further dental treatment

Category	Presence of IF p-value	Presence of IF maxillary sinus p-value	Presence of IF nasal p-value	Presence of IF bone p-value	Presence of IF cystic p-value	Presence of IF dento-alveolar/teeth p-value	Presence of IF soft-tissue calcifications p-value	Need for further dental treatment p-value
Gender								
Female	*P* = 0.70	*P* = 0.25	*P* = 0.41	*P* = 0.87	*P* = 0.71	*P* = 0.76	**P = 0.004**	*P* = 0.52
Male	OR = 1.14	OR = 0.78	OR = 0.75	OR = 1.07	OR = 0.64	OR = 0.93	OR = 2.67*	OR = 0.87
Age	**P = 0.0002**	**P = 0.007**	*P* = 0.24	*P* = 0.22	*P* = 0.42	P = 0.12	*P* = 0.93	*P* = 0.18
< 40 years	**OR**_**1**_ = 3.09*	**OR**_**1**_ = 4.08*	OR_1_ = 0.84	OR_1_ = 0.45	OR_1_ = 0.24	OR_1_ = 2.00	OR_1_ = 0.97	OR_1_ = 1.84
40–60 years	**OR**_**2**_ = 5.37*	**OR**_**2**_ = 5.24*	OR_2_ = 1.75	OR_2_ = 0.87	OR_2_ = 0.53	OR_2_ = 2.26	OR_2_ = 1.12	OR_2_ = 2.33
> 60 years	OR_3_ = 1.73	OR_3_ = 1.28	OR_3_ = 2.08	OR_3_ = 1.93	OR_3_ = 2.22	OR_3_ = 1.13	OR_3_ = 1.16	OR_3_ = 1.26
Tooth status								
Partially edentulous	*P* = 1.00	*P* = 0.85	*P* = 0.36	*P* = 0.25	*P* = 1.00	**P = 0.01**	*P* = 1.00	**P = 0.003**
Fully edentulous	OR = 1.05		OR = 1.65	OR = 1.82	OR = –^a^	OR = 0.41*	OR = 1.04	OR = 0.19*
FOV	**P < 0.0001**	**P < 0.0001**	**P < 0.0001**	**P < 0.0001**	*P* = 0.48	**P = 0.005**	**P < 0.0001**	**P = 0.0002**
Small	**OR**_**1**_ = 4.85*	**OR**_**1**_ = 2.41*	**OR**_**1**_ = 11.02*	OR_1_ = 2.35	OR_1_ = 1.26	OR_1_ = 1.72	**OR**_**1**_ = 41.95*^b^	OR_1_ = 1.24
Medium	**OR**_**2**_ = 22.39*	**OR**_**2**_ = 6.15*	**OR**_**2**_ = 20.81*	**OR**_**2**_ = 8.59*	OR_2_ = 2.64	**OR**_**2**_ = 2.55*	**OR**_**2**_ = 177.66*^b^	**OR**_**2**_ = 3.55*
Large	OR_3_ = 4.59	**OR**_**3**_ = 2.56*	OR_3_ = 1.90	**OR**_**3**_ = 3.66*	OR_3_ = 2.10	OR_3_ = 1.48	**OR**_**3**_ = 4.28*	**OR**_**3**_ = 2.85*

Level of significance set at *p* ≤ 0.05 marked as bold

Statistical analysis regarding the presence of incidental findings and need for further dental treatment in relation to age, gender, tooth status and FOV. Odds ratio (OR) with comparison of 2nd and 1st category (OR_1_), 3rd and 1st category (OR_2_), 3rd and 2nd category (OR_3_) in the order of presentation

*OR significantly different from 1

^a^Values not reliable since calculation contains divisions/multiplications by zero (OR is 0 and/or Infinity)

^b^Only 1 small FOV out of 226 with soft-tissue calcification incidental findings

In patients over 60 years of age, incidental findings were detected in 86% of the CBCT scans. Whereas in patients under 40 years of age, incidental findings were detected in 54% (Table 3). The patient’s age had a significant influence on the occurrence of any incidental finding in the respective CBCT scan (Table 4). Patients older than 60 years were 5.37 times more likely to show any incidental finding in the CBCT examination than patients younger than 40 years (*p* = 0.0002). Significant differences between age groups were also found for incidental findings regarding the maxillary sinus with a 5.24 higher risk for the presence of an incidental finding in the maxillary sinus in patients older than 60 years compared to patients younger than 40 years (*p* = 0.007) (Table 4).

In the dentoalveolar/teeth subgroup, fewer incidental findings were found in fully edentulous patients than in partially edentulous patients (OR = 0.41, *p* = 0.01). However, no significant difference between partially and fully edentulous patients was found for any other or all incidental finding groups (Table 4).

CBCT scans were divided into three groups according to the dimension of FOV with 226 (56%) CBCT scans having a small FOV, 120 (30%) a medium FOV and 58 (14%) a large FOV (Table 1). In CBCT scans with large FOV, in 98% of the examinations at least one incidental finding was found. Whereas in scans with small FOV, at least one incidental finding was found in 72% (Table 3). FOV groups could be confirmed as a risk factor for any incidental finding in multivariate logistic regression (*p* < 0.0001). CBCT scans with large FOV were 22.39 times more likely to show an incidental finding than scans with small FOV. Furthermore, in large FOV, significantly more incidental findings were found than in small FOV for the subgroups maxillary sinus, nose, bone, soft-tissue calcifications (all with *p* < 0.0001) and dentoalveolar/teeth (*p* = 0.005) (Table 4).

Further dental treatment of the patient was radiologically evaluated as needed in 31% (*n* = 126) due to an incidental finding found in the respective CBCT scan (Table 2). FOV size had a significant impact on whether a patient was judged to need further dental treatment and this was 3.55 times more likely in large than in small FOV (*p* = 0.0002). Partial edentulism and large FOV could be shown as risk factors for the need for further dental treatment in multivariate logistic regression (*p* = 0.0003 and *p* < 0.0001, respectively). Further referral of the patient based on incidental findings was judged as indicated in 5% (*n* = 19) (Table 2).

Intraexaminer agreement for any incidental finding was high (kappa = 0.944), indicating an excellent agreement. The highest type-wise kappa values for intraexaminer agreements were reached for nasal incidental findings and soft-tissue calcification (both kappas = 1.000), the lowest regarding the bone (kappa = 0.878). Interexaminer agreement was lower than intraexaminer agreement, but with a kappa value of 0.805 still excellent. Group-wise kappa for incidental findings was highest in the maxillary sinus (kappa = 0.918) and lowest for nasal incidental findings (kappa = 0.668).

## Discussion

In the present study, incidental findings in implant pre-treatment examination CBCT scans were assessed in different FOV and the need for further dental treatment was investigated. In CBCT scans with large FOV significantly more incidental findings were found than in CBCT scans with small FOV (OR = 22.39), and patients older than 60 years were more likely to show an incidental finding than patients younger than 40 years (OR = 5.37). Further dental treatment due to incidental finding was evaluated as needed in 31%, with a higher probability in CBCT scans with large FOV (OR = 3.55). More cases with the need for further dental treatment were identified in partially edentulous patients, compared to fully edentulous patients in the respective FOV.

At least one incidental finding was found in 82% of the scans in this study. This result is consistent with the high rates of incidental findings found in previous studies. In a systematic review performed on the discovery of any incidental finding in CBCT and including 10 studies evaluating scans of the head and neck area, Dief et al. found a positive rate between 24.6 and 94.3% [7]. In a study with a sample of 318 CBCTs acquired, similar to our study for pre-implant planning, but including only large FOVs (13 × 13 cm), at least one incidental finding was found in 93.4% [6]. At least one incidental finding was identified in 91.9% of large FOV (16 × 22 cm) CBCTs in another study that assessed the frequency of nondental pathologies in 1002 patients X-rayed for pre-implant treatment examination [12]. In comparison to these previous studies with large FOV CBCT scans, the study sample of the present study was different, with 56% small FOV. While in CBCT scans with a small FOV, we found a 72% positive rate for incidental findings, the positive rate for incidental findings increased to 98% in large FOV, which is in a similar range to the previously mentioned works.

The most frequent localization of incidental findings in the literature is the maxillary sinus [6, 7]. In line with this finding, the most common incidental finding in the present investigation was a thickening of maxillary sinus mucosa (basal flat or dome-shaped or circumferential flat or irregular thickening) detected in 32% of the scans. Similarly, a positive rate of 29.8% for a thickening of maxillary sinus mucosa was found in an investigation on incidental findings in 300 CBCT scans for implant treatment planning (FOV: 17 × 14 cm) [17]. An even higher positive rate of 55.4% for maxillary sinus mucosal thickening was detected in a study that examined incidental findings in 691 CBCT scans (FOV: 17 × 12 cm) [27]. The higher positive rate of maxillary sinus mucosal thickening in that work may partly be explained by the large FOVs and a different boundary value of mucosal thickening of > 1 mm compared to > 2 mm in our work. Maxillary sinus mucosal thickening > 3 mm was likewise the most frequently detected incidental finding with a rate of 62.6% in a comparable study that evaluated 500 CBCT scans of the maxilla with medium FOV (6 × 6 cm) acquired for dental implant planning [28]. The two previously mentioned studies with higher positive rates of maxillary sinus mucosa thickening included only CBCT scans imaging the maxillary sinus. In contrast, in our study, in 31% only the mandible was imaged. This explains the lower rate of incidental findings in the maxillary sinus in our study.

CBCT scans of patients over 60 years of age had significantly more incidental findings in the present study. Likewise, in their systematic review, Dief et al. found a trend for more incidental findings in older populations [7]. Typical radiologic incidental findings described in older persons in other studies are vascular calcifications increasing with age [6]. In another study, besides the increase of incidental findings with age, an intracranial occurrence of 33.3% of vascular calcification as an incidental finding in CBCT scans was noticed [29]. These findings would probably entail a referral for further diagnostic clarification. In our study, no vascular calcification was detected and a low rate of referrals (5%) due to incidental findings was detected. An explanation is that only 58 large FOV scans were included in the present study and the maximal dimension of a FOV was 14 × 10 cm. Thus, the critical localizations were not imaged. The lack of incidental findings in the vertebral region as well as the very rare occurrence of incidental findings in the temporomandibular joint region in the present work can be explained by the same reason.

Besides the presence of incidental findings in CBCT scans, it is of particular importance whether incidental findings detected in CBCT scans have a therapeutic relevance. Incidental findings with therapeutic relevance in CBCT scans acquired for implant pre-treatment examination could lead to necessary pretreatments or even changes in the therapy plan (Fig. 1). In the present study, the occurrence of incidental findings with therapeutic relevance was high with every third CBCT scan (31%) presenting at least one therapeutically relevant incidental finding. This result stands in line with a recently published study investigating the clinical relevance of incidental findings in 374 CBCT for different indications (51.1% implantology), that found a need for further dental and non-dental therapy in 38.6% of the CBCT [21]. FOV size and tooth status of the patients had a significant influence on the occurrence of incidental findings that needed further dental therapy in our sample. Similar results were found in the previously mentioned work [21]. Furthermore, in CBCT scans for implant planning more therapeutically relevant incidental findings were found than in other indications [21].

By far, the incidental finding with the most severe consequences is a malignancy. In the present study, no malignant tumor was found. In CBCT scans with larger FOV, the potential detection of malignant tumors increases, as the skull base, sinonasal cavities, and other areas adjacent to the maxillomandibular region are included in the FOV [30]. In previous studies, frequencies of malignancies as incidental finding in CBCT ranged from 0.3% (3 of 1000 patients and 3 of 1002 patients) to 1.4% (11 of 795 patients) [12, 31, 32]. Although malignant tumors as incidental findings are rare in CBCT, they are of fundamental importance. If tumors are discovered at an early stage, the prognosis of the patient’s life can be improved if therapy is initiated immediately. Therefore, it is important that clinicians that evaluate CBCT scans are also familiar with possible rare incidental findings and anatomical structures beyond maxillomandibular regions. If a clinician is unfamiliar with a suspicious finding in a CBCT, it is mandatory to seek an opinion from an expert, such as an oral and maxillofacial radiologist [16].

In consistence with the rarely occurring malignant findings, most incidental findings requiring further diagnostic or therapeutic intervention in the present work could be further assessed or treated by a dentist (Figs. 1, 3, 4). Apical lesions occurred in 18% as an incidental finding in the CBCT scans and frequently required mostly endodontic or surgical treatment (Fig. 1). As the two observers in this study had profound surgical experience, many surgically complex treatments were judged treatable by a dentist. Furthermore, the dentists that referred the patients for acquiring a CBCT could be assumed to have profound surgical expertise and clinical experience as they were active in the field of dental implantology. This is an explanation why not a single case was judged to be referred to a maxillofacial surgeon. On the other hand, 19 cases required further evaluation by an otorhinolaryngologist (Fig. 2). This correlates with the frequent occurrence of incidental findings in the paranasal sinus cavities [7, 27, 28].

There are several limitations to the present study. First, as the referring dentist defined the imaged region and patients were not re-examined clinically by the radiologist, the FOV might have been selected larger than necessary. This could have led to a higher incidence of incidental findings. Moreover, the maxillary sinus was imaged relatively often in the study sample as the maxilla was included in 69% of the CBCT scans. Thus, the incidental findings could be overestimated too, since incidental findings are frequent in the maxillary sinus [6, 7, 27, 28].

Another limitation is that the two observers had no access to previously acquired radiographs and clinical findings. Diagnoses were made based on radiological findings only and radiological diagnoses could not be confirmed by further evaluations such as biopsies. Therefore, the classification to need further dental treatment can only be considered as a suggestion based on radiological findings and not as an absolute therapy indication. A final decision on appropriate treatment need can only be made considering clinical findings that were assessed prior to radiological diagnostics by the referring dentist. These data were not accessible to the observers of the present work.

Two experienced dentists in CBCT diagnostics and reporting independently assessed the CBCT scans with an excellent intra- and inter-examiner agreement. However, no other colleagues from related disciplines were included in the study that could have had an impact on the results of the study outcomes. Especially in the area of paranasal sinus findings, a CBCT evaluation of otorhinolaryngologists might have changed the results of the incidental findings and the judgment on the need for further treatment [23]. Many incidental findings that needed further clarification were classified to need further referral to an otorhinolaryngologist. This can be explained by the high prevalence of incidental findings in the paranasal sinus and due to the healthcare system in Switzerland, with otorhinolaryngologist and maxillofacial surgeons’ areas overlapping.

An indication justifying the acquisition of a CBCT is always required [33]. If a CBCT is necessary for implant treatment planning, the ideal FOV has to be chosen according to the patient’s specific situation. The need for further treatment of incidental findings was with 31% considerable in the present study and most of these incidental findings were found in anatomic regions within the dentist's therapeutic range and in scans of partially edentulous patients. These findings underline the importance that the pre-implant assessment must include a complete clinical examination and available radiographs must be consulted. Furthermore, as demonstrated in other studies, uncertain findings in 2D radiographs can be clarified in CBCT [34, 35]. All these factors have to be implicated in the decision of the FOV dimension in CBCT acquisition for implant pre-treatment planning.

## Conclusion

Based on the findings of the present study, it can be suggested that dentoalveolar regions not x-rayed recently are included in the FOV of the CBCT in pre-implant planning and large FOV selected in elderly patients. This may lead to the detection of significant incidental findings that require further therapy or even a change in implant planning, in order to further enhance the quality in diagnostics and treatment.

## Data Availability

The data sets used and analyzed during the current study are available from the corresponding author on reasonable request.
